# An Extended Kriging Method to Interpolate Near-Surface Soil Moisture Data Measured by Wireless Sensor Networks

**DOI:** 10.3390/s17061390

**Published:** 2017-06-15

**Authors:** Jialin Zhang, Xiuhong Li, Rongjin Yang, Qiang Liu, Long Zhao, Baocheng Dou

**Affiliations:** 1 Center for Global Change Studies, College of Global Change and Earth System Science, Beijing Normal University, Beijing 100875, China; tozjlmail@163.com (J.Z.); lixh@bnu.edu.cn (X.L.); zhaolong_ak@163.com (L.Z.); doubc@radi.ac.cn (B.D.); 2 State Key Laboratory of Remote Sensing Science, Jointly Sponsored by Beijing Normal University and Institute of Remote Sensing and Digital Earth of Chinese Academy of Sciences Beijing Normal University, Beijing 100101, China; 3 Chinese Research Academy of Environmental Sciences, Beijing 100012, China; yangrj@craes.org.cn

**Keywords:** wireless sensor network, Kriging interpolation, soil moisture, spectral variables

## Abstract

In the practice of interpolating near-surface soil moisture measured by a wireless sensor network (WSN) grid, traditional Kriging methods with auxiliary variables, such as Co-kriging and Kriging with external drift (KED), cannot achieve satisfactory results because of the heterogeneity of soil moisture and its low correlation with the auxiliary variables. This study developed an Extended Kriging method to interpolate with the aid of remote sensing images. The underlying idea is to extend the traditional Kriging by introducing spectral variables, and operating on spatial and spectral combined space. The algorithm has been applied to WSN-measured soil moisture data in HiWATER campaign to generate daily maps from 10 June to 15 July 2012. For comparison, three traditional Kriging methods are applied: Ordinary Kriging (OK), which used WSN data only, Co-kriging and KED, both of which integrated remote sensing data as covariate. Visual inspections indicate that the result from Extended Kriging shows more spatial details than that of OK, Co-kriging, and KED. The Root Mean Square Error (RMSE) of Extended Kriging was found to be the smallest among the four interpolation results. This indicates that the proposed method has advantages in combining remote sensing information and ground measurements in soil moisture interpolation.

## 1. Introduction

The use of wireless sensor networks (WSNs) is a novel technique for ground data collection that is currently in high demand. It has been applied in various fields, such as hydrology, soil environment, atmospheric environment, forest meteorology, and fire disasters [1]. A WSN is a cross-discipline technology that integrates sensors, automatic control, communication, and data analysis [2]. In contrast to traditional ground data observation methods, the multiple sites comprising a WSN facilitate measurement of regional distribution of parameters. Further, its small size and relatively low price [1] enable more nodes to be installed in the study area to meet research requirements. This helps us to obtain more extensive and much denser observation data of a specific land surface parameter. Compared with satellite remote sensing techniques, WSNs are a more flexible platform that can support a larger variety of sensors [2,3]. For example, they can measure leaf area index (LAI), soil moisture, soil temperature, electrical conductivity, and atmospheric infrared radiance temperature at each observation node [4,5]. In contrast to remote sensing, in which data are obtained during overpass periods, monitoring by WSN is continuous and in real time. This complements the area data measured by remote sensing and also provides reference data for validation of satellite and aerial remote sensing [6].

However, WSNs only measure point data, whereas spatially continuous data over a certain region are increasingly required in environmental researches and management in order to make effective decisions or justified interpretations [7]. Consequently, to determine the spatial distribution of a land surface parameter, data interpolation is necessary. Some traditional interpolation methods, such as the inverse distance weighting method (IDW), polynomial method, and Kriging method, have been widely used to convert point data to spatial distribution. The IDW method and the polynomial method are relatively fast and easy to compute, but can’t explore the spatial variation of relations between nodes and have limited estimation accuracy [8]. In contrast, the Kriging method uses variogram analysis to estimate the spatial variation structure, and takes spatial autocorrelation into consideration [9,10]. The Kriging method is a group of stochastic interpolation methods comprising the Simple Kriging method, Ordinary Kriging (OK) method, Universal Kriging method, Co-kriging method, Regression Kriging method, and Residual Kriging method [11]. These methods have been used in various studies. For example, Maruyama et al. [12] estimated peak ground velocities using the Simple Kriging method. Wu et al. [13] utilized the Residual Kriging method with the input variables of latitude, longitude, and elevation to estimate the average monthly temperature in the United States. Liang et al. [14] used the Co-kriging method to estimate daily NO_3_-N loads in an agricultural river with the assistance of daily discharge. Cantet [15] used the Kriging with external drift (KED) method to map the mean monthly precipitation of a small island located in the Lesser Antilles. Through the development of geostatistical interpolation methods, more information about the rule or pattern of the distribution of the target parameter was explored, either through statistics of this parameter, or through its correlation with other parameters.

Soil moisture is a vital factor in agriculture, ecology, and hydrological studies [16,17,18,19]. Dynamic monitoring of soil moisture on the scale of irrigation fields is an essential part of precision agriculture. Despite advances in recent interpolation algorithms, estimation of the spatial distribution of near-surface soil moisture is still unsatisfactory because soil moisture is highly heterogeneous on both the spatial and temporal scales, even over short distances [20]. Many researchers have applied remote sensing to invert soil moisture. For example, Fan et al. [21] improved the performance of Ts/VI space in retrieving soil moisture based on Compact Airborne Spectral Imager (CASI)/Thermal Airborne Spectrographic Imager (TASI) data. El Hajj et al. [22] used neural networks (NNs) to estimate surface soil moisture from X-band SAR data over irrigated grassland areas. Ponnurangam et al. [23] used a compact polarimetric decomposition with surface component inversion to estimate soil moisture on bare and vegetated agricultural soils. However, soil moisture imposes significant difficulty in quantitative remote sensing inversion because optical remote sensing is not directly sensitive to soil moisture, whereas thermal infrared and passive microwave remote sensing usually have low spatial resolution. Although radar has the capability to provide high spatial resolution, in order of tens of meters, they are more sensitive to surface roughness, topographic features and vegetation, which means that the soil moisture inversion process is very difficult. Ground observation data, such as that from WSN measurement, are always necessary as supplementary data for remote sensing inversion of soil moisture. Conversely, remote sensing information can be viewed as supplementary data to aid the interpolation of ground-measured soil moisture. In this light, Kang et al. [24] upscaled the WSN-measured soil moisture via Regression Kriging method with the aid of multi-resource remote sensing information over heterogeneous cropland. Liao et al. [25] analyzed various sources of uncertainty, such as soil properties and terrain indices, while estimating near-surface soil moisture content with the aid of Co-kriging at two typical hill slopes. However, as soil moisture is affected by many factors, such as elevation, vegetation, temperature, and irrigation, it is rather difficult to single out one significantly linear correlated factor to aid interpolation. Some researchers have also tried to use data assimilation methods in soil moisture estimation. Gao et al. [26] estimated the spatial pattern of soil moisture using the Bayesian maximum entropy (BME) method, which is based on WSN data and auxiliary information from ASTER (Terra) land surface temperature measurements.

In this paper, we propose extending the Kriging method by introducing high-resolution remote sensing imagery spectral variables into the interpolation algorithm. The algorithm was analyzed and compared with OK, Co-kriging and KED algorithms, and then applied to the soil moisture data acquired by WSN in the HiWATER campaign to generate time series of soil moisture maps at 30 m spatial resolution and daily temporal resolution.

## 2. Materials and Methods

### 2.1. Study Area

**Figure 1:**
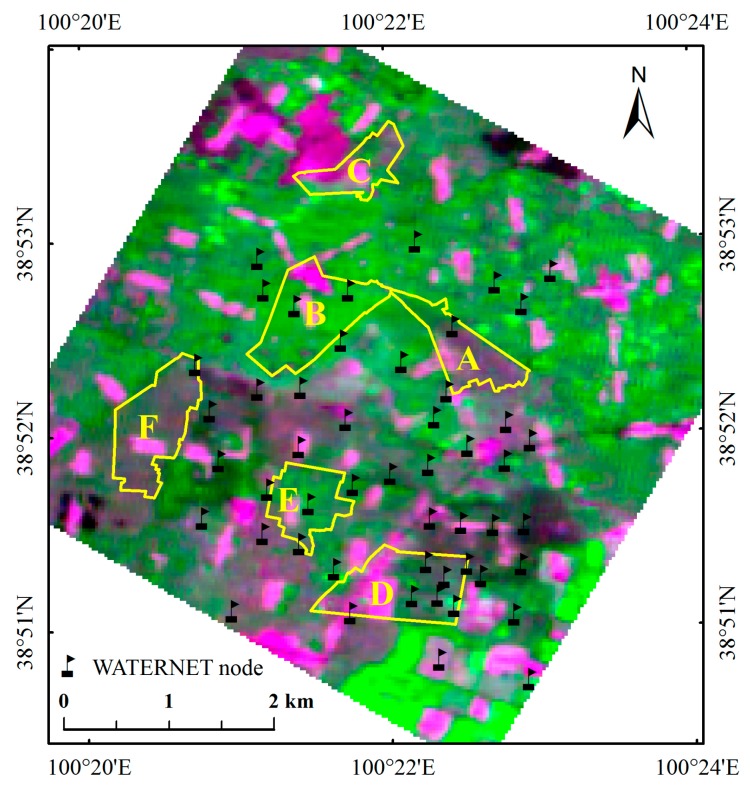
Study area in the middle reaches of the Heihe River Basin: the image was obtained from HJ satellite, combined by band 3 (RED), band 4 (NIR) and band2 (GREEN); the numbered yellow polygons indicate the selected irrigation fields in study area: A is JC-6, B is SQ-1, C is STZ-1, D is WX-4, E is XM-2, and F is ZH-6.

The study area, shown in Figure 1, is located in the Zhangye oasis in the middle reaches of the Heihe River Basin (HRB) in northwestern China. The HRB is the second largest inland river basin and is characterized by large areas of alpine cold and arid landscapes and a small portion of oasis agricultural land. The potential evaporation ranges from 1200 mm to 1800 mm per year, and the average annual precipitation in the artificial oasis is about 177 mm. Irrigation is the primary source of water for crops.

The HRB has long served as a test bed for integrated watershed studies and hydrological experiments [27]. HiWATER is an ongoing watershed eco-hydrology comprehensive experiment that began in 2012 [28]. With the objective of improving the comprehensive observation ability, an eco-hydrological WSN was installed as a part of the HiWATER basic experiment. From May 2012 to September 2012, 50 WATERNET nodes were installed in a 5.5 km × 5.5 km foci experimental area in the main oasis in the middle reaches. In this study, we chose an area of approximately 4.5 km × 5.0 km as the experimental area, which consists of 48 WATERNET nodes (Figure 1). All the nodes were installed in cornfields.

### 2.2. Data Resource

WSN data and remote sensing data were both used in this study. We used the data covering the period from 10 June 2012 to 15 July 2012, when the vegetation cover of the study area changed from sparse to dense. As the main crop in this area is maize, which has a great portion of roots distributed in 0~15 cm zone [29] and depends much on irrigation, monitoring of near-surface moisture is needed for the study of irrigation management. There were 48 WATERNET nodes deployed in the study area to measure soil moisture and soil temperature in two layers (4 cm, 10 cm), and the data were collected every 5 min [30]. In this study, we used the WSN-measured soil moisture at 10 cm depth for interpolation. The soil moisture measurements are based on the frequency-domain reflectometry method using a Hydro Probe II (HP-II) sensor [26]. Details of WATERNET design and other information are given in Jin et al. [31]. Because the data collected by some of the WSN nodes are affected by sensor noise and other abnormal conditions during wireless data transfer, smoothing and noise reduction treatments are necessary. Besides, when the soil moisture is close to saturation, the soil moisture sensors cannot work properly and sometimes give abnormal values [32]. Thus, this part of data also need to be removed. We first excluded the abnormal data by assigning the zero value, negative value and abnormally high value (soil moisture content >50%) as invalid data NaN. Then, we averaged the data for the whole day, and used the average result as the final soil moisture value for each node.

Remote sensing data served as auxiliary information in estimating the near-surface soil moisture of the study area. The CCD cameras onboard the Chinese HJ microsatellite (HJ-CCD) provide remote sensing observations of the study area in four spectral bands (blue, green, red, NIR), with spatial resolution of 30 m and revisiting frequency of approximately 2 days. The HJ-CCD images were geometrically and radiometrically preprocessed. The georectification was carried out with automatically matched control points using a Level-1TP Landsat5 TM image as reference, and the residuals of control points were less than 0.5 pixel. Calibration was conducted with coefficients in the metadata of HJ-CCD images, and atmosphere correction was carried out via 6s atmospheric model with daily aerosol and water vapor parameters acquired by a CIMEL CE318 sunphotometer deployed in the study area during the campaign of HiWATER.

Two remote sensing variables, NDVI (Normalized Difference Vegetation Index) and albedo, were used in this research. They were derived from pre-processed HJ-CCD image as follows:

(1) $$NDVI=\frac{\rho_{4}-\rho_{3}}{\rho_{4}+\rho_{3}}$$

(2) $$albeo=-0.5878\rho_{1}+0.6543\rho_{2}+0.4183\rho_{3}+0.38914\rho_{4}$$

where $\rho_{1}$ to $\rho_{4}$ stands for the surface reflectance in the four spectral bands, respectively, and the coefficients of Equation (2) are obtained from Gao et al. [33].

**Figure 2:**
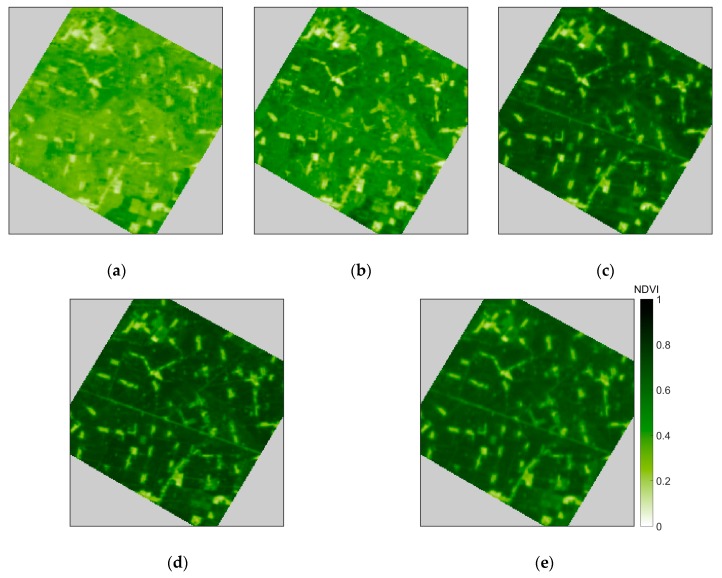
Five available NDVI maps from 10 June 2012 to 15 July 2012: (**a**) is on 15 June; (**b**) is on 19 June; (**c**) is on 29 June; (**d**) is on 8 July; and (**e**) is on 13 July.

Owing to the influence of clouds, only five clear-sky images on the following dates during this period could be used (shown in Figure 2): 15 June, 19 June, 29 June, 8 July, and 13 July in the year of 2012. As the interpolation was applied from 10 June 2012 to 15 July 2012, we made a daily linear interpolation of the NDVI and albedo for the Kriging calculation.

**Figure 3:**
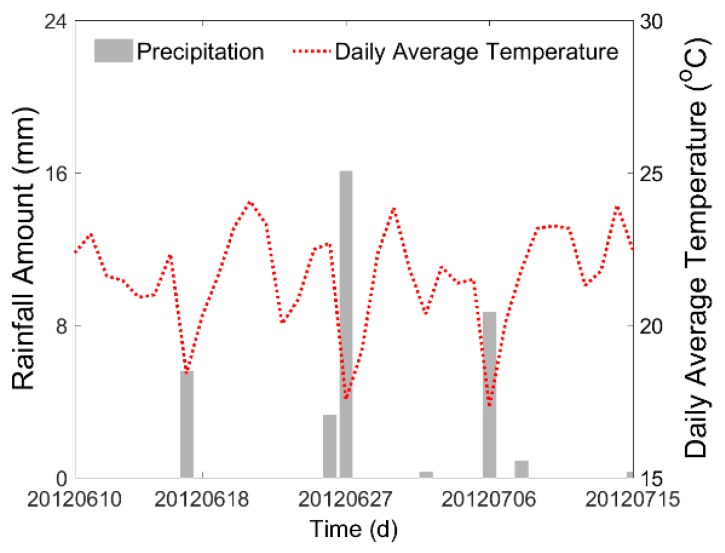
Precipitation condition and daily average temperature of the study area from 10 June 2012 to 15 July 2012.

The meteorological data were acquired in the Daman Superstation [34,35] which is near the center of the study area. Figure 3 shows the precipitation and daily average air temperature of the study area. The irrigation data used were provided by the Cold and Arid Regions Sciences Data Center at Lanzhou (http://westdc.westgis.ac.cn). Although the records were not complete (some omissions exist), the available parts were still sufficient to meet the demand in this study. Six typical irrigation fields were chosen from the study area and their soil moisture estimation results were compared with the precipitation and irrigation data. The names and locations of these irrigation fields are shown in Figure 1: Jincheng-6 (JC-6), Shiqiao-1 (SQ-1), Shang Touzha-1 (STZ-1), Wuxing-4 (WX-4), Xiaoman-2 (XM-2), and Zhonghua-6 (ZH-6).

In this study, we introduced a soil moisture map as simulation data in calculating the semivariogram and some interpolation analysis. This soil moisture map was derived from the airborne sensors of CASI/TASI data [21] with spatial resolution of 3 m.

### 2.3. Method

The new spatial interpolation method proposed in this paper is based on the Ordinary Kriging algorithm. The proposed method extends the traditional X and Y spatial coordinates to spatial and spectral combined coordinates, and utilizes remote sensing derived spectral variables NDVI and albedo in the interpolation algorithm as supplementary information. In this section, first, an outline of the traditional Kriging algorithm is given, then the technique employed to extend the Kriging algorithm is explained. While fitting the semivariogram of the soil moisture, as the number of WSN nodes is insufficient to gather robust statistics, we used the remote sensing-derived soil moisture map mentioned in Section 2.2 to calculate the variance function.

#### 2.3.1. Traditional Kriging Method

(a) Basic Formula

Kriging is an interpolation method derived from regionalized variable theory, which inherited the concept from geostatistics [11]. It has been used to provide linear unbiased predictions at unsampled locations and depends on expression of the spatial variation of the variable in terms of the semivariogram [36,37]. This method quantifies and reduces the uncertainties of estimation, minimizing self-estimated prediction errors [26]. The core of Kriging is an optimally linear unbiased estimator that can be expressed as follows [38]:

(3) $$Z^{*}\left(v_{0}\right)=\overset{n}{\underset{i=1}{\sum }}\lambda_{i}Z\left(v_{i}\right)$$

where $Z^{*}$ is the estimated value of the variable at location ${\;v}_{0}$, *n* is the number of the closest neighboring sampled data points used for interpolation, $\lambda_{i}$ is the Kriging weight assigned to each observation${\;Z(v}_{i})$.

Optimal estimation requires the minimum variance of errors:

(4) $$\sigma_{\kappa }^{2}=\mathrm{Var}\left[Z\left(v_{0}\right)-Z^{*}\left(v_{0}\right)\right]=E\left\{{\left[Z\left(v_{0}\right)\;-\overset{n}{\underset{i=1}{\sum }}\lambda_{i}Z\left(v_{i}\right)\right]}^{2}\right\}=\min$$

To ensure unbiased estimation, the following constraint must satisfy the equation as follows:

(5) $$\overset{n}{\underset{i=1}{\sum }}\lambda_{i}=1$$

To solve this constrained optimization problem, the Lagrange Multiplier Method (LMM) is adopted. With Equation (4) as the objective function and Equation (5) as the constraint, the LMM minimizes the following cost function:

(6) $$f\left(\lambda_{1}{,\;\lambda }_{2},\;\cdots {,\;\lambda }_{n},\;\mu \right)=\frac{1}{2}\;E\left\{{\left[Z\left(v_{0}\right)\;-\;\overset{n}{\underset{i=1}{\sum }}\lambda_{i}Z\left(v_{i}\right)\right]}^{2}\right\}+\mu \left(1\;-\;\overset{n}{\underset{i=1}{\sum }}\lambda_{i}\right)$$

where *μ* is the Lagrange Multiplier. At the minimum point of the cost function, the differentiation of *f* with respect to each of its variables is zero. Thus, the optimization problem decomposes into one of solving the following set of equations:

(7) $$\{\begin{array}{c} \frac{\partial f}{\partial \lambda_{i}}=0,\;i=1,\;2,\cdots ,n,\\ \frac{\partial f}{\partial \mu }=0, \end{array}$$

Differentiating the cost function, we have:

(8) $$\{\begin{array}{c} \frac{\partial f}{\partial \lambda_{i}}=\lambda_{i}E[Z^{2}(v_{i})]+\underset{j\neq i}{\overset{n}{\underset{j=1}{\sum }}}\lambda_{j}E[Z(v_{i})Z(v_{j})]-E[Z(v_{0})Z(v_{i})]-\mu =0,\\ \frac{\partial f}{\partial \mu }=1-\overset{n}{\underset{i=1}{\sum }}\lambda_{i}=0, \end{array}$$

If we know $E\left[Z^{2}\left(v_{i}\right)\right]$, $E\left[Z\left(v_{0}\right)Z\left(v_{i}\right)\right]$, and $E\left[Z\left(v_{i}\right)Z\left(v_{j}\right)\right]$, then the equations can be solved. These values are estimated by the semivariogram function in Section 2.3.1 (b).

The minimum variance of error ($\sigma_{\kappa }^{2}$), as is shown in Equation (4), can be used as a quality indicator in estimation [39]. It can evaluate the intrinsic estimation of uncertainty from the algorithm itself.

(b) Estimating Semivariance and Semivariogram

Semivariance and semivariogram, containing spatial correlation information, are important concepts in geostatistics. The semivariance of variables at certain locations is estimated from the semivariogram function, which is a function of the distance between the two locations. Usually, a de-trending pre-process is applied to the observation data. After this pre-processing, the spatial distribution of the variable is assumed stationary, which means that the semivariance does not change with location. On the basis of this assumption, the semivariance can be estimated from the data that a random variable is well correlated in space as a function of separation distance. The semivariance (*γ*) of *Z* between two data points is defined as:

(9) $$\gamma \left(x_{i},x_{0}\right)=\gamma \left(h\right)=\frac{1}{2}\mathrm{Var}\left[Z\left(x_{i}\right)-Z\left(x_{0}\right)\right]$$

where *h* is the distance between points $x_{i}$ and $x_{0}$, and γ(h) is the semivariogram [40].

The semivariogram is usually estimated from the statistics of sample points as follows:

(10) $$\hat{\gamma }\left(h\right)=\frac{1}{2n}\overset{n}{\underset{i=1}{\sum }}{\left(Z(x_{i})-Z(x_{i}+h)\right)}^{2}$$

where *n* is the number of pairs of sample points separated by distance *h* [41].

**Figure 4:**
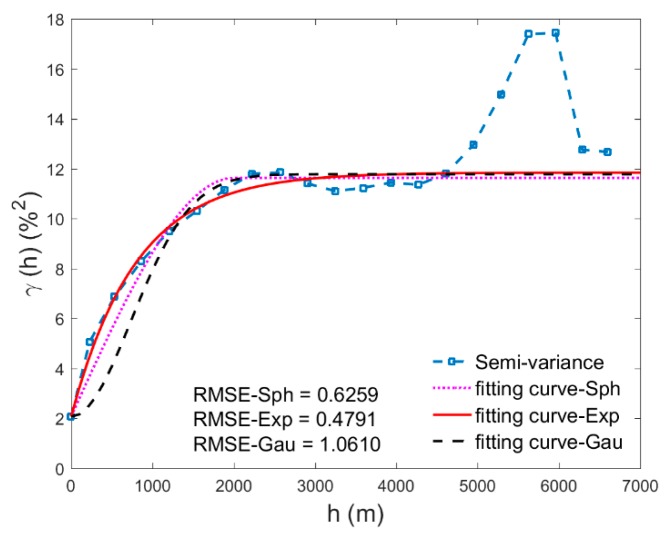
Semivariance of sampled soil moisture data and the fitting curves of the soil moisture semivariogram: the semivariance was calculated by the 9000 random sampling points from the soil moisture map; the semivariogram was fitted by the Spherical model, Exponential model, and Gaussian model, with the data of *h* ≤ 4000 m, and the range was set as 2400 m.

As the number of WSN nodes is insufficient to gather robust statistics, the soil moisture map retrieved from airborne hyperspectral remote sensing was used here to derive the semivariogram function. In the calculation, we sampled 9000 random points from the soil moisture map and excluded those points on residential area. Before calculating the semivariance, spatial trend should be removed and stationarity should be checked [42]. In consideration of trend, we linearly fitted the spatial trend and removed it from the data, and finally used the residuals for the calculation of the experimental variogram. The spatial distance was divided into twenty bins, and the average semivariance of point-pairs in each bin was derived to represent the semivariance value of the bin (shown in Figure 4).

Three commonly used semivariogram models: Spherical (Sph) model, Exponential (Exp) model, and Gaussian (Gau) model, were compared here to find the optimal model for semivariogram fitting. The model equations are shown in Equations (11)–(13):

(11) $$\gamma {\left(h\right)}_{Sph}=\{\begin{array}{c} C_{0},\;h=0\\ C_{0}{+C}_{1}\left[1.5*\left(\frac{h}{a}\right)\;-\;0.5*{\left(\frac{h}{a}\right)}^{3}\right]\;,\;0\leq h\leq a\\ C_{0}{+C}_{1},\;h>a \end{array}$$

(12) $$\gamma {\left(h\right)}_{Exp}=\{\begin{array}{c} C_{0},\;h=0\\ C_{0}{+C}_{1}\left[{1\;-\;e}^{-\frac{h}{a}}\right],\;h>0 \end{array}$$

(13) $$\gamma {\left(h\right)}_{Gau}=\{\begin{array}{c} C_{0},\;h=0\\ C_{0}{+C}_{1}\left[{1\;-\;e}^{-{\left(\frac{h}{a}\right)}^{2}}\right],\;h>0 \end{array}$$

where γ(h) is the semivariance; $C_{0}$ represents a nugget, which is the minimum variability observed or the “noise” at a distance of zero; $C_{1}$ is the structural variance, $C_{0}{+C}_{1}$ represents the sill variance; and *a* is the range that signifies the correlation length in geostatistics.

As the amount of the sampling data was insufficient when the spatial distance was beyond a certain extent, its average semivariance value was not stable. These invalid data should be removed before semivariogram fitting. Therefore, we only used the data when *h* ≤ 4000 m in the fitting process. As suggested by Minasny and Mcbratney [43] that the nugget can be estimated as the semivariance at the shortest possible separation distance, we fix the nugget to the average semivariance at our shortest lag of 30 m, which takes the value of 2.0747. Fitting lines of these three models shown in Figure 4, together with the RMSE (Root Mean Square Error), evaluate the fitting performance of the models. As can be seen from Figure 4, Exponential model estimated the semivariance curve better than the other two models, and its RMSE value, 0.4791, is also the smallest. So, we finally chose Exponential model (with parameters $C_{0}=2.0747$, $C_{1}=9.7697$, a = 2400 m) as the semivariogram model. Here, we assumed that this semivariogram could be applied to interpolate WSN measured soil moisture in the period from 10 June 2012 to 15 July 2012.

(c) Traditional Kriging Method with Auxiliary Variables

There are several existing methods which incorporate auxiliary information in interpolation in the frame of Kriging theory, for example, the Co-kriging method and KED method. All these algorithms operate on spatial space when introducing the auxiliary variables.

The Co-kriging method estimates the objective variable via the easy observable variables by using their correlation [44], whereas the correlation between the objective variable and auxiliary variables is implied. Specially, when the number of auxiliary variables is low and they are not available at all grids in the study area, Co-kriging can be used to improve the interpolation prediction. KED method originated in petroleum and gas exploration [45]. Instead of using monomials of the coordinates in Universal Kriging method, its drift is defined externally through the auxiliary variables [46,47]. It is necessary to stress that the auxiliary variables incorporated in the form of an external drift should be highly linearly correlated with the variable of interest [45].

In this study, in order to test the reasonability for the interpolation result of the proposed Extended Kriging method, we used Co-kriging method and KED method as contrast in the following analysis.

### 2.3.2. Selection of Spectral Variables

**Figure 5:**
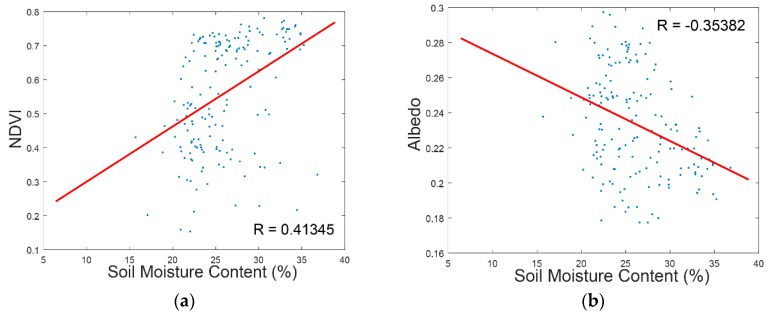
Correlation analysis between auxiliary variables (NDVI and albedo) and soil moisture: (**a**) the NDVI data was derived from the five available HJ satellites images and compared with the soil moisture data observed by WSN of the corresponding dates; (**b**) the albedo data was derived from the five available HJ satellites images and compared with the soil moisture data observed by WSN of the corresponding dates.

In this study, we selected NDVI and albedo as the auxiliary information to aid the interpolation. It is based on the following three main considerations: (1) NDVI and albedo can reflect surface soil moisture variation directly or indirectly [48,49]; (2) NDVI and albedo are the spectral indexes which are fairly easy to be obtained from almost all high resolution remote sensing data sources; (3) NDVI and albedo represent most of the remote sensing information in the visible and near infrared spectral range. Although selecting NDVI and albedo as spectral variables is out of practical consideration, it is still necessary to analyze the correlation between soil moisture and NDVI/albedo, which are shown in Figure 5.

In Figure 5a,b, the NDVI and albedo data were collected from five available HJ satellites images to compare with the soil moisture data observed by WSN of the corresponding dates. As can be seen from the comparison results, the NDVI and albedo are correlated to soil moisture to a certain extent, but the correlation coefficient is not significant (absolute value less than 0.5). An explanation of this result may be that NDVI can reflect the vegetation status, and vegetation usually grows good when soil moisture is abundant [50]. However, in irrigated land, all the fields get enough irrigation; then the correlation between soil moisture and NDVI are weakened. In sparsely vegetated land, the albedo of dry soil is usually higher than that of wet soil [48]. Therefore, there is a correlation between soil moisture and albedo, but this correlation can be disturbed by soil type and the presence of vegetation. Actually, it is usually considered impossible to estimate soil moisture from visible and near infrared remote sensing data.

### 2.3.3. Extending the Kriging Method to Incorporate Remote Sensing Information

To reflect more details of the spatial distribution pattern of soil moisture, we propose a new algorithm that incorporates remote sensing variables, i.e., NDVI and albedo, into the basic Kriging method. The traditional interpolation space is the spatial space depicted by *x* and *y* coordinates. The new algorithm extends the interpolation space to the combined spatial and spectral space, in which NDVI and albedo are treated as coordinates, just like x and y. The distance in the combined space is characterized by the spatial distance and the spectral distance, as follows:

(14) $$h=\sqrt{{\Delta x}^{2}{+\Delta y}^{2}}$$

(15) $$s=\sqrt{{\left(\frac{\Delta \mathrm{NDVI}}{\sigma_{\mathrm{NDVI}}}\right)}^{2}+{\left(\frac{\Delta \mathrm{albedo}}{\sigma_{\mathrm{albedo}}}\right)}^{2}}$$

where *h* is the spatial distance, Δx and Δy are the coordinate differences between two sampled points; *s* represents the spectral distance, ΔNDVI and Δalbedo are the differences of NDVI and albedo values between two sampled points, and $\sigma_{\mathrm{NDVI}}$ and $\sigma_{\mathrm{albedo}}$ are two normalization factors (in this study, we simply set their values as 0.1, 0.1).

**Figure 6:**
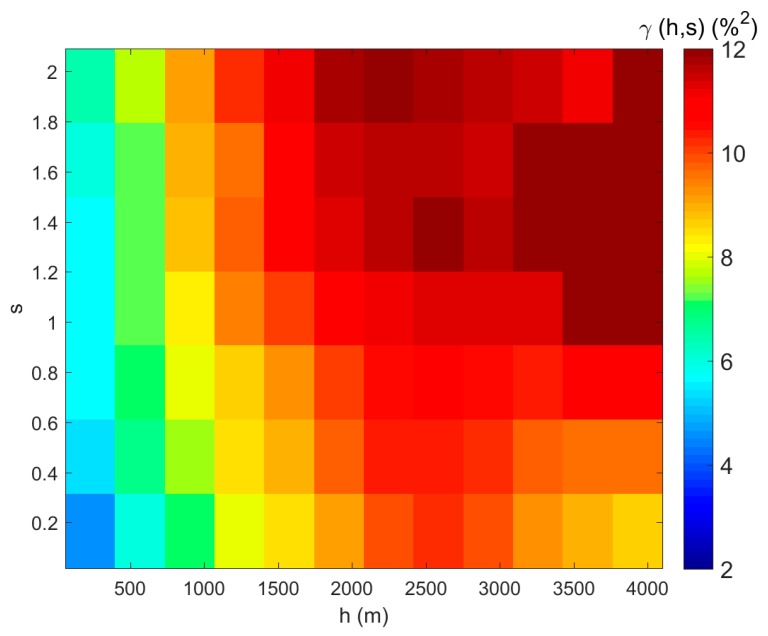
Semivariance of sampled soil moisture data with respect to spatial and spectral distance: the *X*-axis is spatial distance, the *Y*-axis is spectral distance, and the color in each grid represents the average semivariance value of the soil moisture; only valid semivariance data were kept for the semivariogram fitting (*h* ≤ 4000 m and *s* ≤ 2).

Correspondingly, the semivariance calculation and the semivariogram model was extended to the spatial and spectral combined space. We also used the soil moisture map to derive the semivariance statistics, and the de-trending process was also done before the calculation. The spatial and spectral distance were divided into twenty bins respectively. The bin values with insufficient sample numbers were removed before semivariogram fitting. We only used the data when *h* ≤ 4000 m and *s* ≤ 2 in the fitting process. The semivariance, as a function of *h* and *s*, is shown in Figure 6. We estimated the nugget at our shortest lag of 30 m, and shortest spectral lag of 0.005, which is millesimal of the largest spectral distance. The nugget $\left(C_{0}\right)$ value is 1.8875.

In fitting the semivariogram, we also compared Spherical (Sph) model, Exponential (Exp) model, and Gaussian (Gau) model, and calculated their RMSE values to choose the proper one. As the RMSE value of Exponential model is 0.5612, smaller than the others (RMSE_Sph_ = 0.6312, RMSE_Gau_ = 0.9054), we also used the Exponential model for the semivariance fitting. The extended Exponential model is represented in the equations below:

(16) $$\gamma_{1}\left(h\right){=C}_{1}\left[{1\;-\;e}^{-\frac{h}{a_{1}}}\right],\;h>0$$

(17) $$\gamma_{2}\left(s\right){=C}_{2}\left[{1\;-\;e}^{-\frac{s}{a_{2}}}\right],\;s>0$$

(18) $$\gamma \left(h,\;s\right){=\gamma }_{1}\left(h\right){+\gamma }_{2}\left(s\right){+C}_{0}$$

where $\gamma_{1}$ and $\gamma_{2}$ are the semivariogram values with respect to *h* and *s*, *γ* is the overall semivariogram, and $a_{1}$ and $a_{2}$ are the lag distances of the spatial and spectral variables.

**Figure 7:**
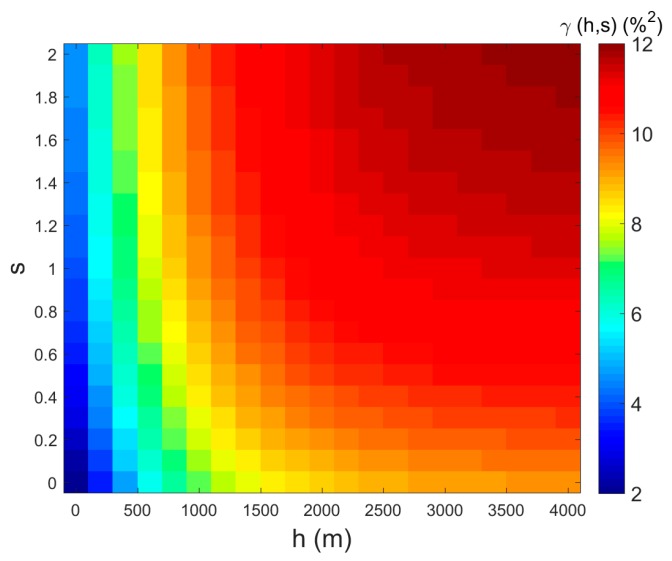
Fitting result for soil moisture semivariogram in the spatial and spectral dimensions, using the Exponential model: the *X*-axis is spatial distance, the *Y*-axis is spectral distance, and the color in each grid represents the average semivariance value of the soil moisture.

Using the semivariogram model as in the above Equations (16)–(18), the fitting semivariance diagram can be obtained, as shown in Figure 7. The $a_{1}$ of spatial distance was pre-set as 2400 m, and the $a_{2}$ of spectral distance was pre-set as 2.5. Then, the fitted parameters ${\;C}_{1}$, and ${\;C}_{2}$ were 7.3909, and 2.9696, respectively.

## 3. Results and Discussion

The proposed Extended Kriging method was applied to the WATERNET measurements in the HiWATER campaign to generate daily soil moisture maps in the 4.5 km × 5 km oasis area from 10 June to 15 July 2012. To evaluate the performance of Extended Kriging method, we first visually inspected the interpolation results and compared them with the results obtained using OK method, Co-kriging method and KED method. Then, a quantitative uncertainty analysis was conducted with the RMSE from leave-one-out cross-validation method. Finally, we interpolated the soil moisture distribution for every day from 10 June 2012 to 15 July 2012, and examined the precipitation and irrigation data to ascertain whether the temporal variation of the interpolated soil moisture was consistent with the water input.

### 3.1. Spatial Trend Analysis and Cross Validation

**Figure 8:**
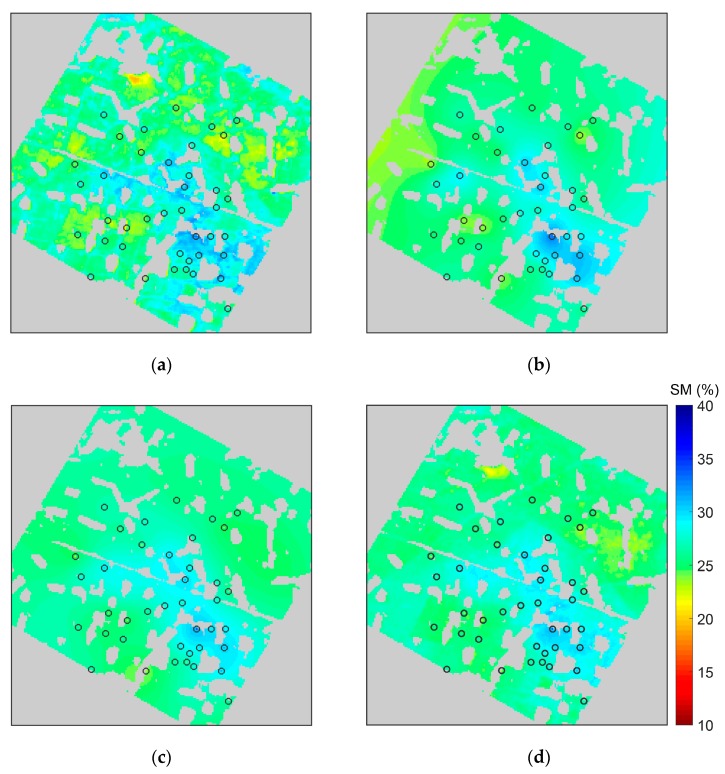
Interpolated soil moisture (SM) at depths of 10 cm on 10 July 2012, using WSN measured data: (**a**–**d**) are respectively the results of the Extended Kriging, OK, Co-kriging, and KED methods; the circles in maps represents the WATERNET nodes.

A spatial map of the interpolated soil moisture can directly reflect the capability of the interpolation methods to obtain the parameter’s variation pattern. We used the semivariogram obtained from Section 2.3, soil moisture data (45 nodes) of the WATERNET at depth of 10 cm on 10 July 2012, and the remote sensing data (NDVI and albedo) on 10 July 2012, obtained by linear interpolation, to estimate the soil moisture distribution of the experimental area. The Extended Kriging and OK interpolations were implemented by MATLAB language; Extended Kriging interpolated with the aid of NDVI and albedo, and OK interpolated using only WSN data. The Co-kriging and KED interpolations were implemented with the GSTAT package in R programming language with the aid of NDVI and albedo. The interpolation results of these four methods are shown in Figure 8a–d, which are respectively the results of the Extended Kriging, OK, Co-kriging, and KED methods. All result maps are masked to exclude residential areas. It is clear that the result for the Extended Kriging method shows more details of the field soil moisture distribution than that for OK and Co-kriging methods. The interpolation results for OK and Co-kriging methods can only show smooth and continuously changing trend surfaces (Figure 8b,c), while the interpolation results for Extended Kriging method (Figure 8a) reveals a few rough veins in the ground surface and subtle changes in the field soil moisture. Although the result from KED method (Figure 8d) shows some heterogeneous features, it is still not as evident as the result from our Extended Kriging method.

The leave-one-out cross-validation method was adopted to estimate the interpolation accuracy. In the leave-one-out cross-validation method, every sample serves as a testing sample; assuming that the number of samples is N, then there are N−1 training samples each time and, finally, N testing results. The RMSE of these N testing results is used to evaluate the interpolation accuracy. The cross-validation results are shown in Table 1, which shows that the RMSE value of OK is higher than that of Extended Kriging, and is lower than those of Co-kriging and KED.

### 3.2. Temporal Trend Analysis and Correlation with Precipitation/Irrigation Data

**Figure 9:**
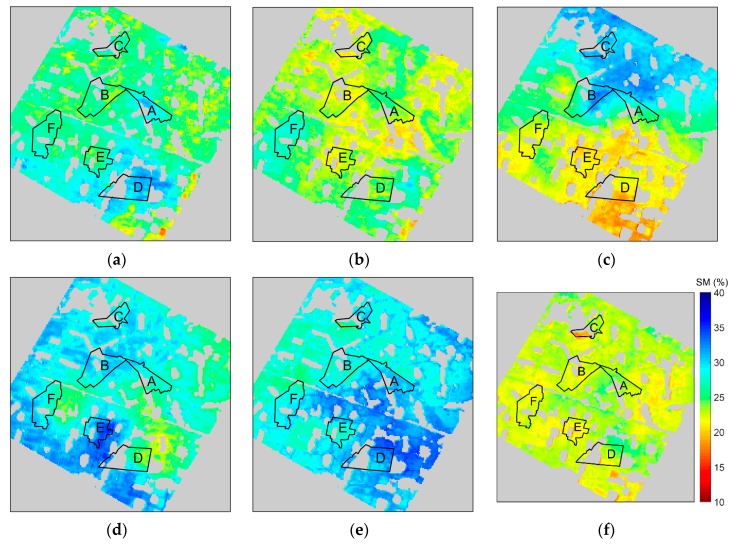
Interpolated soil moisture (SM) maps by Extended Kriging method in the study area: the subfigures corresponds to the dates of 10 June (**a**), 17 June (**b**), 24 June (**c**), 1 July (**d**), 8 July (**e**) and 15 July (**f**), 2012, respectively; six irrigation fields are shown in maps: A is JC-6, B is SQ-1, C is STZ-1, D is WX-4, E is XM-2, and F is ZH-6 .

In addition to measuring data from multiple sites simultaneously, WSN is also able to obtain real-time and continuous observations. This enabled us to estimate the continuous soil moisture distribution of the study area on both spatial and temporal domains. We interpolated the soil moisture distribution for every day from 10 June 2012 to 15 July 2012. Consequently, we obtained 36 interpolation maps. Due to malfunctions of some WSN nodes, the average number of valid WSN nodes could be used in this period is about 40, ranging from 36 to 45. Six of the maps, with seven-day intervals, are shown in Figure 9. As can be seen, the spatial distribution of the soil moisture content changed significantly over time, indicating the necessity for dynamic monitoring of soil moisture. The drought areas and their borders can be clearly identified in the interpolated maps, which is very useful information for agricultural management.

**Figure 10:**
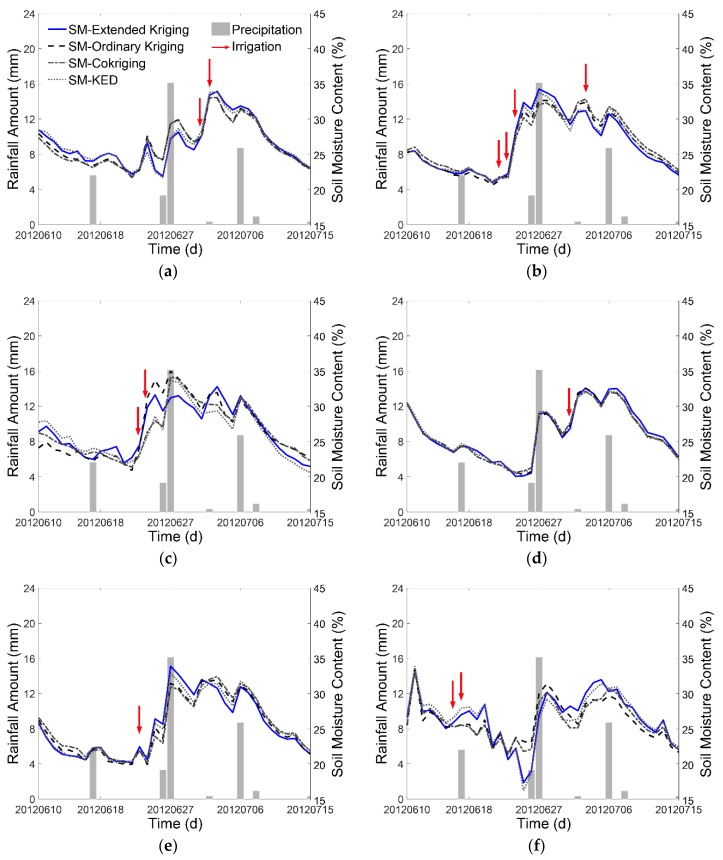
Comparison of soil moisture (SM) changing trend with precipitation and irrigation data of the chosen irrigation fields: (**a**) JC-6; (**b**) SQ-1; (**c**) STZ-1; (**d**) WX-4; (**e**) XM-2; and (**f**) ZH-6; the grey bar represents precipitation; the red arrow represents the irrigation.

To verify the authenticity of the time series of the interpolated soil moisture map, we chose six irrigation fields in the study area and compared the estimated results of Extended Kriging method and three other methods with the precipitation and irrigation data on temporal series from 10 June 2012 to 15 July 2012. Figure 9 shows the soil moisture spatial distribution of the six irrigation fields. The comparison results are shown in Figure 10. As shown, the precipitation records indicate that there was rain on 17 June, 26 June, 27 June, 3 July, 6 July, 8 July, and 15 July. Furthermore, the irrigation dates are different for different fields. It is clear that the soil moisture value increases in response to the precipitation or the irrigation, and then gradually decreases as the fields dry out.

**Figure 11:**
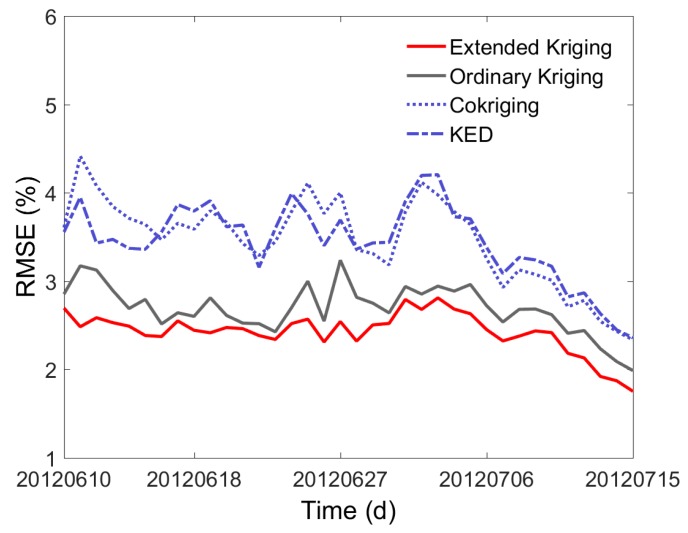
Comparison of the RMSE calculated by the Extended Kriging, OK, Co-kriging, and KED.

The leave-one-out cross-validation was also used for accuracy evaluation. Figure 11 is the time series of the average RMSE of all available WSN nodes, derived from the leave-one-out method. As is seen from Figure 11, the RMSE values of OK are higher than that of Extended Kriging, and are lower than those of Co-kriging and KED. This means that in condition of the weak correlation between soil moisture and the two remote sensing variables, our Extended Kriging method can make use of the two indirectly related variables to improve the estimation of soil moisture distribution, while the Co-kriging and KED methods didn’t perform that well. This indicates that our Extended Kriging method is more applicable when the correlation between object variable and auxiliary variable is relatively weak.

## 4. Conclusions

With the rapid development of ground-based Earth observing techniques such as wireless sensor network, we are now able to monitor environmental parameters in real time, continuously, and with multiple sample points. However, interpolation is still needed to extend the point measurement to spatial distribution of the corresponding parameter in an area. As satellite remote sensing is an efficient way of acquiring area Earth observing data, it is desirable to combine information from remote sensing and from ground-based observation networks.

The Extended Kriging method proposed in this study introduces the remote sensing image spectral information into the traditional interpolation method. NDVI and albedo are the spectral variables used in the algorithm. These spectral variables are treated in the same manner as the spatial variables, i.e., x and y. Therefore, the interpolation is fundamentally the same Kriging algorithm, but operating on the combined space of spatial dimension and spectral dimension. The semivariogram model is also extended to the combined space. A remote sensing derived soil moisture map is used in this paper to fit the semivariogram model. However, this soil moisture map can be replaced by other sources of samples as long as the dataset is sufficiently large to derive robust statistics about the semivariance.

The proposed algorithm was applied to the soil moisture dataset acquire by the soil moisture sensors network (WATERNET) in the oasis agricultural areas, which is the foci experimental area of the HiWATER campaign. As the WATERNET provides continuous near-surface soil moisture measurement over 48 scattered points, the interpolation results are daily soil moisture maps from 10 June 2012 to 15 July 2012, covering an area approximately 4.5 km × 5.0 km in size. Visual inspections indicate that the interpolation result from the proposed Extended Kriging algorithm presents much more spatial details than that of the OK and Co-kriging algorithms, and more contrast than that of the KED algorithm. The field-average soil moisture of several irrigation fields for long time series are associated with the precipitation data and irrigation data, and the temporal variation of soil moisture can be well explained by these water inputs. The quantitative uncertainty analysis with the leave-one-out method indicates that the Extended Kriging algorithm is more applicable than Co-kriging and KED algorithms when the correlation between object variable and auxiliary variable is relatively weak. Currently, NDVI and albedo are recommended as the spectral variables to aid interpolation because they can be easily derived from most high-resolution satellite images. However, we demonstrated in the discussion that more relevant spectral variables, such as LST, could be incorporated into this algorithm to improve its performance. However, how to choose the informative spectral variables remains an open topic for this algorithm.

Although some results from the Co-kriging and the KED are presented, we prefer not to compare the proposed algorithm with other sophisticated algorithms in terms of accuracy for the following two considerations: in the first place, the Extended Kriging algorithm is much simpler than the Co-kriging, the KED, and the data assimilation method. Thus, it is possibly applicable in situations where the pre-conditions of other sophisticated algorithms are not satisfied. Second, as a new algorithm, the Extended Kriging still needs improvements. For example, the normalization factors for spectral variables could be refined, and the semivariogram model in the combined space is too simple. Nevertheless, we prefer to present the simplest form of the algorithm to the reader, and leave these improvements to future researchers.

There are other aspects that we could not discuss in more depth in this short paper. One of them is the quality, or accuracy, of WSN-measured soil moisture. As we know, it is technically difficult to install Hydro Probe II (HP-II) sensors exactly at 4 cm and 10 cm below the surface; and the WSN sensors are more or less affected by its internal voltage and temperature; and the sensors can go to saturation in extremely low or high soil moisture values [26,32]. It is also true that the remote sensing data may suffer from inaccurate calibration and atmospheric correction. Besides the flaws of data, the scale mismatch between the footprint of WSN nodes and 30 m resolution remote sensing pixels should be considered. Instead of deriving semivariogram from field measurements, the semivariogram in this study comes from a soil moisture map which is inverted from hyperspectral remote sensing image. The scale difference between these two kinds of semivariogram is subtle and may bring extra uncertainty in the interpolation of WSN measurements. Nevertheless, currently we do not have solid data to support analysis on these aspects.

Another related interesting topic is the desired density of the WSN nodes, and how to optimize the location of the nodes. Fortunately, a similar topic has been addressed by other researchers [51], although their research area and target parameter are different. In this paper, we simply used all the valid WATERNET data.

The proposed algorithm is a development of the classical Kriging method. Although it is proposed in this paper to interpolate the soil moisture data, it is potentially applicable to other environmental parameters. As new observation technologies are being applied wider, more and more high-quality measurement data, at multiple sites in a small area, will become available to the public, and the potential of the Extended Kriging can be further explored.

## Figures and Tables

**Table 1:** Comparison of the interpolation results for the Extended Kriging method, OK method, Co-kriging method, and KED method on 10 July 2012.

Method	$\sigma_{k}$ (%)	RMSE (%)
Extended Kriging	2.7684	2.4185
Ordinary Kriging	2.9234	2.6218
Co-kriging	2.9438	3.0110
KED	2.9454	3.1686
